# Is there a generalized magnitude system in the brain? Behavioral, neuroimaging, and computational evidence

**DOI:** 10.3389/fpsyg.2013.00435

**Published:** 2013-07-15

**Authors:** Filip Van Opstal, Tom Verguts

**Affiliations:** Department of Experimental Psychology, Ghent UniversityGhent, Belgium

Some 10 years ago, it was proposed that different quantities, including time, space or number, are processed by a common mechanism (Walsh, 2003; Pinel et al., 2004; Cohen Kadosh et al., 2005), henceforth called generalized magnitude system (GMS). This presented an important attempt at theoretically integrating data across various literatures, and became very influential (e.g., Burr et al., 2010; Lourenco and Longo, 2011; Bonato et al., 2012). However, in this paper we will evaluate the concept in a critical way. We first argue that such a mechanism faces conceptual difficulties. Second, we look at empirical findings that were proposed to support a GMS and will offer a different explanation of these findings based on computational modeling and recent empirical observations. Third, we review positive evidence in favor of distinct magnitude mechanisms. We discuss alternatives for a GMS in a final paragraph.

A first difficulty a GMS faces is the metric to code different types of magnitudes. What common metric allows comparing the loudness of a tone to the duration of a stimulus or the meaning of an Arabic digit? In other words, what is the scale factor that relates one magnitude dimension to another? One strong interpretation of the GMS would imply that there is a single common currency on which all dimensions can be mapped. Early studies addressed this issue for one particular pair of dimensions, namely event counts and time (duration). A scale factor of 1:5 was obtained, in which the representation of one count is equivalent to 200 ms of time (Meck and Church, 1983). However, it was demonstrated that a single scale factor fails to explain all observations (e.g., Balci and Gallistel, 2006). Furthermore, numerical effects on the magnitude of grip aperture are flexibly modulated by the relative magnitude of the numbers on a specific trial (Chiou et al., 2012). In other words, the scale factor by which one magnitude is mapped to another can change on a trial-by-trial base. To fit the framework of a GMS, the common metric thus has to be sensitive to the relative magnitude in a specific context. This flexibility of the scaling factor violates the prediction from a GMS for a common monotonic mapping of quantities (Bueti and Walsh, 2009). Another difficulty for a common monotonic mapping concerns metathetic dimensions, i.e. magnitude dimensions that lack an intrinsic polarity (Stevens, 1957). What is, for example, the larger magnitude when judging luminance? If luminance is defined with respect to black, increasing darkness then increases the magnitude. On the other hand, if luminance is defined with respect to white, magnitude should decrease with increasing darkness. Another issue concerns the specific problem the brain faces when representing numbers. From a computational point of view, representing numbers is inherently different from representing other magnitude dimensions, such as luminance, surface area, or weight. A problem specific for processing numerosity is that it requires the individuation of objects independent of their non-numerical features. How the brain represents numbers is therefore likely to depend on a unique set of neural properties. These unique properties to encode discrete numerosities could be the reason why differences in processing between discrete and continuous magnitudes have been observed (Castelli et al., 2006).

Concerning the second argument, much of the evidence in favor of a GMS comes from the observation that behavioral effects can be very similar across stimulus types, or that they interact with each other. One important behavioral effect that has often been used to argue along these lines is the comparison distance effect (CDE). The CDE indicates a decrease in response times when the magnitude between two stimuli that need to be compared increases (Moyer and Landauer, 1967). The presence of a CDE when comparing different types of stimuli, such as numbers, luminance, weight and even social status, has led to the suggestion that the representations of these stimuli must overlap (e.g., Moyer and Landauer, 1967; Chiao et al., 2004). However, as we have demonstrated before (Verguts et al., 2005; Van Opstal et al., 2008) the CDE can originate from the pattern of connections from input coding neurons to the decision and response neurons, relevant for the magnitude comparison task (e.g., “Left stimulus is larger”). In particular, the CDE may result from a competition in the decision process that is determined by the monotonically increasing pattern in these connection weights (see Van Opstal et al., 2008 for full details). In fact, this connectivity pattern automatically develops from the requirements of a magnitude comparison task, and a CDE will thus be observed for any stimulus type trained on this task. Therefore, the presence of a CDE across stimulus types does not imply a common representation or dedicated magnitude processing system. Instead, similar effects may arise from similar computational constraints across tasks. For a similar argument in the context of the size congruity effect, see Santens and Verguts (2011).

Similarly, if the CDE does not originate from stimulus representation, brain activity that correlates with the CDE is not necessarily caused by the activation of the stimulus representation. Rather than revealing a common representation for different stimulus types (e.g., Pinel et al., 2004; Cohen Kadosh et al., 2005), this activity could be related to decisional or comparison processes (e.g., Gobel et al., 2004). The observation that different types of stimuli evoke a CDE in the same brain area does therefore not argue for a shared magnitude representation.

Third, recent investigations have revealed positive evidence against a GMS. A GMS implies a shared neuronal substrate for magnitude. It is commonly assumed that the intraparietal sulcus (IPS) is the most likely candidate for a shared neuronal space because of its strong involvement in number processing (for reviews see Dehaene et al., 2003; Cohen Kadosh et al., 2008). However, neural recordings in monkey IPS by Tudusciuc and Nieder (2007) revealed no correlation between neurons' tuning for analog and discrete quantities. Also, across length and duration tasks, Genovesio et al. (2012) found no correlation in magnitude tuning in macaque prefrontal neurons. However, they did find a very strong correlation across length, duration, and matching tasks for the task-relevant feature (e.g., cells preferring “Choose green stimulus” in one task also preferred it in another task), suggesting that these neurons coded decision/response processes rather than abstract magnitude. Similar results were obtained with functional magnetic resonance imaging (fMRI) studies in humans. In one study, a discrete/analog response task was used to investigate if analog and discrete quantities are processed in the same brain area (Castelli et al., 2006). In this task, participants have to judge whether there is more green or blue on a display. The amount of green and blue could vary continuously (analog) or in discrete areas in time or space. Results revealed that different areas of the IPS were involved for analog and discrete quantities for both temporal and spatial conditions. Others found that the IPS is not activated more during non-symbolic numerosity processing than during a same-different color discrimination task that does not involve magnitude (Shuman and Kanwisher, 2004). This indicates that IPS is not specifically activated for the processing of non-symbolic numerosities. As noted by these authors, a coherent theory of magnitude processing in IPS that includes symbolic number and other magnitudes (e.g., lines, angles, and luminances) but that excludes the assessment of non-symbolic numerosity is difficult to imagine. Transcranial magnetic stimulation (TMS) of the IPS revealed a similar distributed pattern of magnitude processing. Stimulation of the left IPS slowed down performance in a numerosity comparison task, whereas left (or right) IPS stimulation did not affect duration comparison (Dormal et al., 2008). Dormal and colleagues showed a similar dissociation between duration and number processing in non-demented Parkinson's disease patients: Whereas the patient group suffered from a decrease in performance compared to controls in a duration task, this was not the case in a numerosity comparison task (Dormal et al., 2012). Recent research also demonstrated impaired time perception in children with difficulties in numbers and mathematics, but failed to show a correlation between timing performance and mathematical abilities (Hurks and Van Loosbroek, 2012; Vicario et al., 2012). Here, the interaction between time and numbers was attributed to differences in the internal clock (Hurks and Van Loosbroek, 2012), or to differences in attention or sensitivity (Vicario et al., 2012). An interesting pattern of results was demonstrated by Rusconi et al. (2006) who found interactions between pitch height and space in trained musicians independent of the relevance of pitch height in the task. In non-trained musicians, however, an interaction was only observed when both pitch height and space were relevant for the task. When only an implicit reference was made for pitch height comparisons, no interaction was observed. This suggests a necessity to explicitly encode stimuli to obtain interaction effects in this population. This is also witnessed by the flexibility of the mapping from numbers to space (e.g., Fischer et al., 2010; Van Dijck and Fias, 2011) demonstrating that the monotonic coding between magnitude dimensions is not as straightforward as suggested by a GMS.

To deal with these conceptual and empirical problems, weakened versions of a GMS and alternative proposals have been put forward. One suggestion is that different magnitude representations are processed separately, but interact at later decisional or motor stages. Separate dimension specific representations might share a decision procedure or a common comparison process (e.g., Feigenson, 2007; Chiou et al., 2012; Dewind and Brannon, 2012; Vicario, 2013). This echoes with our suggestion that the CDE is related to decision processes and is therefore observed whenever the magnitude of two stimuli is compared (Van Opstal et al., 2008; see also Cohen Kadosh et al., 2008). Previous work demonstrated that the interaction between different magnitude dimensions, such as space and numbers, can also originate from similar interactions at the decision stage (Gevers et al., 2006). In line with this, it was recently proposed that the observed interaction between random movement generation and the perception of numbers (Daar and Pratt, 2008; Vicario, 2012) could be caused by an early competition between decisional processes (Vicario, 2012). An alternative but not mutually exclusive suggestion is that the mapping of different magnitudes onto each other occurs in working memory because of task demands. The importance of how stimuli are encoded in working memory was recently investigated for the interaction between numbers and space. When participants were asked to retain a series of numbers in memory while they were performing a go/no-go parity judgment task, Van Dijck and Fias (2011) showed that the relation between numbers and space depended on the serial encoding of the number stimuli in working memory rather than the magnitude of the numbers. The observation that an interaction between different types of magnitude is only present when they are part of the task context (Cappelletti et al., 2011) or in trained experts (Rusconi et al., 2006) could also point to the need to actively encode stimuli in working memory.

In sum, although there is a bulk of evidence showing crosstalk and commonalities between magnitude dimensions, a magnitude system in which these dimensions are monotonically mapped to each other faces many empirical and conceptual difficulties. We propose instead that overlap in computational constraints, working memory, or decision/response processes across quantitative dimensions and tasks may account for observed similarities.
